# The Tea House Series: Striving Together to be Antiracist

**DOI:** 10.1007/s11606-022-07519-z

**Published:** 2022-06-16

**Authors:** Yalda Shahram, Di’Reon Lowry, Nicholas Iverson, Arianne Teherani

**Affiliations:** 1 Department of Medicine, Division of Hospital Medicine, University of California San Francisco, San Francisco, CA USA; 2 University of Illinois College of Medicine at Peoria, Peoria, USA; 3 Department of Medicine, Division of General Internal Medicine, University of California San Francisco School of Medicine, San Francisco, USA

**Keywords:** medical education, continuing education, race/racism, disparities, health equity

## Abstract

**Background:**

Though awareness of health care structures that are racist and oppressive is increasing among health care professionals, there is a gap in continuing education curricula focused on antiracist anti-oppressive practices, and limited faculty and staff development to guide individuals towards action.

**Aim:**

To develop, implement, and evaluate a novel antiracist faculty and staff development program called the Tea House Series.

**Setting and Participants:**

A five-part continuing education series with an accompanying online community for faculty and staff at the divisions of Hospital Medicine in one institution in the western United States.

**Program Description:**

The four foundational pillars integral to the Tea House Series were as follows: educational framework based on the pedagogy of Paulo Freire, local disparities data, welcoming space to establish a community of practice and accountability. Each session contained participant dialogue in small group activities.

**Program Evaluation:**

Qualitative analysis of participant survey responses demonstrated transformation towards a hope to act with a sense of community. Quantitative analysis measured increased confidence for the program learning objectives.

**Discussion:**

The Tea House Series may be used as a model for continuing education to provide the tools and the community to confront systems of racism and oppression in any institution.

**Supplementary Information:**

The online version contains supplementary material available at 10.1007/s11606-022-07519-z.

## INTRODUCTION

Health care disparities have been well documented and are ubiquitous, occurring in high-performing systems with highly motivated, excellent clinicians.^1^ These disparities are often linked to structural oppression, defined as policies, economic systems, and institutions that produce and maintain modern social inequities.^2^ Structural oppression manifests in disparities based on patient social characteristics, such as gender, race, language spoken, geographic location, and socioeconomic status, in health care outcomes, access, and experiences.^1^ Education on dismantling structural oppression is important at all learner levels, and continuing education (CE) on the topics of antiracism, structural racism, critical race theory, and other anti-oppression curricula is needed across the country in health professions institutions.^3^ Given this demand for CE, currently available programs focus primarily on social determinants of health and cultural competency.^4–7^ Despite these well-intentioned curricula, disparities persist because disremembering the context of structural oppression has contributed to its perpetuation by silence and indifference.^8–10^ Establishing the historical context of White supremacy ideology, colonialism, and imperialism, and the current lack of antiracist policies is necessary in any curricula for health care practitioners that aim to address inequity, with an emphasis on unlearning the language of dehumanization and centering the patient.^11–13^ Dismantling structural oppression requires awareness of the problem. Also needed are actions that address oppression, with strategies to confront inequities at the individual and institutional level. Therefore, we sought to develop and evaluate an anti-oppressive curriculum about how to confront inequities while we strive together to be antiracist and provide access to materials and guides for the implementation of the educational program to teach about structural oppression.

## SETTING AND PARTICIPANTS

The Tea House Series for faculty and staff in the University of California San Francisco (UCSF) Divisions of Hospital Medicine (DHM) was implemented from October 2020 through June 2021 with 50 faculty and staff participating. CE credit was offered at the end of the series. All sessions were conducted virtually due to the COVID-19 pandemic. To provide context for the setting, demographic data for clinicians in the DHM at UCSF Health and for United States Hospital Medicine are compared in Appendix 1 of the ESM. The UCSF Office of Human Research Ethics/Institutional Review Board (OHRE/IRB) reviewed this study and deemed it exempt.

## PROGRAM DESCRIPTION

The Tea House Series was designed with a focus on pragmatic and evidence-based strategies to motivate providers to model anti-oppressive and antiracist behaviors in their own practice and take action to confront structural oppression in their institutions. The Tea House series provides structured sessions with an intentional focus on learners who are practicing health care professionals, such as faculty in academic centers and attending providers in community practices.^14^ “Tea House” in different cultures is a place for community to gather and have dialogue, while drinking tea.^15^ Using this imagery, we invoke a welcoming space to invite participation from learners in a community of practice, which underscores that “community creates the social fabric within which learning occurs”.^16^ The Series consists of five virtual sessions designed using Kern’s Six-Step Curriculum Development for Medical Education model.^17^ Session titles and the learning objectives are outlined in Appendix 2 of the ESM, including session structure, objectives, and literature. Curricular content was updated iteratively in accordance with current events, including the COVID pandemic, and its impact on health care disparities.

Paulo Freire’s pedagogy of critical consciousness is the first foundational pillar integral to the Tea House Series. The conceptual framework for our curriculum was informed by Freire, where we “adopt a method which fosters dialogue and reciprocity, [where] one must first be ideologically committed to equality, to the abolition of privilege, and to non-elitist forms of leadership wherein special qualifications may be exercised but are not perpetuated.”^18,19^ The second foundational pillar calls on local institutional examples of oppression and disparate health care data. The approach of emphasizing historical and political roots of structural oppression using examples from within the local institution and region is the second foundational pillar and a result of critical pedagogy, where we necessitate participants to be curious, to search, to invent and reinvent, and to apply learning to concrete situations.^11,20^ We practice this by asking key dialogue questions that foster engagement and discussion about antiracist actions. At UCSF, we discuss two examples: the death of Ishi and the experimentation on Elmer Allen at UCSF.^21,22^ Elmer Allen was a man from Richmond, CA, who identified as Black and was part of the racist history of medical experimentation. At UCSF in the 1940s, he was injected with plutonium and lost his leg. The highlighting of cases in health care that cause collective and generational trauma illustrates not just racism but a system of oppression. With principles from “How to be an Antiracist”,^23^ the series strives to motivate participants towards action and “radical empathy”.^24^ In the final session of the Series, the *Theatre of the Oppressed* and the forum theater modality allows participants to practice radical empathy, defined as the ability to relate to another from their perspective, not from your perspective, or “putting yourself in someone’s shoes.” This activity permits an understanding of the impact of structural oppression, and challenges participants to propose actions to create change in the narrative. The third foundational pillar is a welcoming space to establish community, for participants and facilitators to interact in dialogue, inviting a diversity of thought, value, and morals. Dialogue is in the form of small and large group activities, discussing questions with guidance and probing from peer facilitators, and treating learners with respect and humility, particularly those from oppressed backgrounds. To provide autonomy to oppressed voices is described by Freire: “to respect the other in dialogue is to work with each other rather than one person acting on the other person.” When a topic has the potential to trigger trauma, as topics in this antiracist series often do, following the framework of Freire with an emphasis on respect can help mitigate harm to the individual, particularly for those who carry traditionally oppressed identities. Feeling uncomfortable was explicitly expected and presented at the start of every session with the “agreement” slide (Appendix 3 of the ESM). To be antiracist is a lifelong journey and after each session, participants are reminded about the fourth foundational pillar integral to the Tea House Series: accountability. The third pillar creates the space for community to form during the sessions of the Tea House, and the fourth pillar creates a community of practice beyond the sessions using an online platform. By asking participants to share S.M.A.R.T. goals as pertains to the session objectives, the online community provides support: in changing the culture at the institution and on the journey to learn, unlearn, teach, and take action to be antiracist.

## PROGRAM EVALUATION

Qualitative analysis to measure transformation and impact on behavior used free text responses, compiled and analyzed by 2-separate coders using thematic analysis, then reconciled through iterative discussion.^25–27^ Themes are outlined in Table 1 with representative quotes. Free text response to the question prompt: “How does seeing the data impact your motivation to take or not to take action?” was analyzed to evaluate critical reflection, dialogue, and action.^25^ The disparities data embedded in the survey question highlighted a racialized gap in 7-day readmission, length-of-stay, and opioid prescribing practice within the respondent’s institution.^28^ The theme of *Data Motivates Action* was expressed by nearly every respondent, exemplified by this quote: “If I see data that shows disparities that my clinical care is contributing towards, that spurns me to take discrete action.” Additional themes emerged when analyzing the free text response to the question prompt: “How do you or do you not feel empowered [to address health care disparities]?” The major theme of “feeling overwhelmed” to address structural racism and systemic change was expressed by respondents in the pre-survey before the first session. One respondent wrote, “I feel like I have very little power as an individual in a large health system”. After the first session, and increasingly throughout the Series, the respondents’ comments fit the themes of “hope to act” and “sense of community”, exemplified by the quote, “I feel empowered by being part of a supportive learning community.” Some respondents also described a theme of “lack of resources” to address institutional or systemic racism, expressions of feeling ill-equipped, lacking time and training.

**Table 1.** Thematic Analysis of Free Text Survey Responses Assessing Empowerment to Address Health Care Disparities and Motivation to take Action for 50 Faculty and Staff Participants Who Complete the Tea House Series at the University of California, San Francisco School of Medicine

Theme	Representative quotes
Feeling overwhelmed	“I feel empowered to address interpersonal and some systemic disparities at the institutional level (e.g., changes to the EHR), but the larger societal inequities and root causes are quickly overwhelming and I feel deeply challenged by how to address broader upstream causes of disparities.”
	“I feel like I have very little power as an individual in a large health system”
	“Systemic disparities are bigger than I can fix”
	“Also, I feel the healthcare system is inherently racist and I am not sure if I am empowered to address big picture systemic racist policies in health care as an immigrant physician who cannot vote.”
Hope to take action	“I feel I can speak up either with colleagues, leadership and learners - particularly if it relates to events or learning/curricula.”
	“I have a better awareness and a slightly better framework to learn about the health care disparities of our patients, but most of all, I need to learn from more examples - not just dramatic examples like discovering the true preferred language of the patient, but small and definite victories. “
	“I feel I can bring these topics up now in the clinical setting and in teaching settings (or both).”
Sense of community	“I feel like I am in an environment that allows for open conversation and the resources that the department has to offer allows me to find avenues to do so.”
	“I feel empowered by being part of a supportive learning community.”
Data motivates action	“If I see data that shows disparities that my clinical care is contributing towards, that spurns me to take discrete action.”
	“The data is compelling, much to discuss and work on. Definitely motivates me to take action.”
	“The data are reliable and compelling. I’m already convinced and am committed to taking evidence-based action.”
Lack of resources	“I feel empowered to recognize though do not feel I have access to resources to make meaningful change for individuals with disparity”
	“Agree because I do think it’s encouraged. not strongly because I don’t feel like there are resources for this and I don’t have enough training”

Quantitative analysis was used with Likert-scale questions assessing participant confidence in each learning objective before and after each session to confirm that the curriculum was achieving its aims. Aggregate pre- and post-session confidence scores were created, demonstrating increased participant confidence in learning objectives after participation (Table 2). Respondents were asked to self-assess their confidence to be able to achieve the learning objectives, and we recognize the limitations of Likert-scale questions, namely susceptibility to response bias. It was encouraging to measure an increase, especially when respondents chose very low confidence pre-session, for example, to practice radical empathy in session five.

**Table 2.** Likert-Scale Questions Assessing Participant Confidence in Each Learning Objective for All 5 Sessions

Confidence in learning objectives: aggregate score		
	Pre-session	Post-session
Far above-average confidence	10.75%	26.26%
Somewhat above-average confidence	30.65%	45.45%
Average confidence	36.56%	2.23%
Somewhat below-average confidence	16.67%	5.05%
Far below-average confidence	5.38%	0.00%

## DISCUSSION

“Striving Together To Be Antiracist” means forming a community to confront and dismantle the structures of oppression together, not by the neoliberal paradigm of individual action, but rather through a paradigm shift to forming community. A “State of Oppression” is more easily maintained with the neoliberal paradigm because it divides the oppressed, and “maintains the oppressed *I* in a position of ‘adhesion’ to a reality which seems all-powerful and overwhelming.”^18^ Silence and inaction are “anti-dialogue” to maintaining the status quo.^19^ Individuals enter the Tea House with feelings of hopelessness to address systemic inequities, coming from a state of anti-dialogue, but quickly learn critical consciousness, with analysis of the context, self-awareness of their potential to transform, increased confidence, and a sense of community which gives them hope to take antiracist action.^20^

Historical and political context cannot be redacted in anti-oppressive curricula, and in the Tea House Series, we show a transformation of participants because of the inclusion of local histories and institutional disparities data. Dismantling structures of oppression requires action, but begins with awareness, reflection, and dialogue. The significant increase in confidence for the learning objectives confirms participant awareness. We achieve reflection and dialogue by interactive activities in each session of the Series. By asking participants to share S.M.A.R.T. goals on the online community platform, we have created a space beyond the sessions of the Tea House for accountability to take antiracist action. Choosing an online platform was in part due to the virtual nature of the Series in the time of the restraints for in-person gathering because of the COVID-19 pandemic. Unforeseen benefits of an online platform included participants’ ability for sharing resources, including books, articles, podcasts, and videos. And an online platform allows access at any time and avoids scheduling conflicts. In the future, we plan to qualitatively analyze the shared S.M.A.R.T. goals by participants of the Tea House Series to study their strategies to confront inequities at the individual and institutional levels.

Limitations of our program include the point that there is no quick fix or bandage that can repair the structures of oppression. We agree with some respondents’ comments on a lack of resources devoted to promoting education on how to be antiracist, coupled with prioritization of the time, funding, restructuring of leadership, and diversifying workforce all hinder the work needed to create anti-oppressive and antiracist institutions and practices in health care. Participation in the Tea House Series was voluntary without compensation for time or effort. Clinical service duties and scheduling conflicts would preclude participation.

Faculty in Academic Centers and attending providers in Community Practices may adopt the Tea House Series to their institutions by substituting their own local histories and disparities data, beginning with the indigenous lands occupied by their health care centers. Consulting with their local community in equal partnership is also key, to learn direct experiences of racism at the institution to inform activities (Appendix 2 of the ESM).

The next step for the Tea House Series is expanding to an interprofessional and multidisciplinary audience at UCSF with participants from the Schools of Dentistry, Pharmacy, Medicine, Nursing, Graduate Division, and Physical Therapy.

## Supplementary information

ESM 1 (DOCX 375590 kb)
