# Supplementary material for: The Tea House Series: Striving Together to be Antiracist
Source: J Gen Intern Med. 2022 Jun 16;37(9):2318–22. doi: 10.1007/s11606-022-07519-z (PMC9202981; doi:10.1007/s11606-022-07519-z)
Supplement: Supplementary file 1 — (DOCX 375590 kb) [file 11606_2022_7519_MOESM1_ESM.docx]

APPENDICES

Appendix 1

Comparison of UCSF Campus DHM vs national Hospital Medicine physician demographics

| UCSF DHM* | | |
| --- | --- | --- |
| **Race/Ethnicity** |  | |
| - White | | 46% |
| - South Asian | | 24% |
| - East Asian | | 16% |
| - Hispanic or Latinx | | 5% |
| - Southeast Asian | | 3% |
| - Black or African American | | 1% |
| - Native Hawaiian or Pacific Islander | | 1% |
| - Declined to state | | 3% |
| **Gender**: Women | | 55% |
| **Sexual orientation**: Heterosexual | | 87% |

*^ UCSF DHM Diversity Taskforce Final Report 2020

| U.S. Hospital Medicine Physicians’ Race/Ethnicity^†^ | |
| --- | --- |
| White | 36% |
| Asian | 37% |
| Hispanic or Latinx | 8% |
| Black or African American | 5% |
| Mixed Race | 4% |

^†^^^ Medscape Hospitalist Compensation Report 2019: [link](https://www.medscape.com/slideshow/2019-compensation-hospitalist-6011429)

Appendix 2

Structure, objectives, and literature used to guide the Tea House Series curriculum at the University of California, San Francisco: (1) Timeline for the Series, (2a) Presentation slides with notes per session, (2b) Facilitator guides for small group activities for each session, (2c) Specific references and further reading per session, (3) Literature used in multiple sessions

(1) Timeline for the Series

(2) Session Content: (2a) Presentation slides with notes per session, (2b) Facilitator guides for small group activities for each session, (2c) Specific references and further reading per session

Note: specifically, we will have a dialogue about local roots

Historical Context and policies are informed by White Supremacy ideology and manifest in health care with disparities data.

Facilitator Guide:

- *Remember the purpose of the dialogue is to achieve the objectives of the session*
- *Remind participants that they can turn off video at any time for any reason and welcome everyone into the dialogue using our Agreement*
- *During introductions, invite participants to share name, site, and how they move through the world (alluding to any social identities they want to name). “my name is Yalda Shahram, I am faculty at DHM, and I move through the world as (identify as) a cis-gender woman, an immigrant, bilingual, heterosexual, person of color.”*
- *During introductions also invite participants to review the Agreement to dialogue presented in the intro slides and to add/modify it if they would like (but do not take more than 2 minutes on this).*

Small Group Activity 1 – a brief history review (25 min)

Material needed: PDF of History Review Activity

*If the participants have not read the history review PDF, then ask them to read Ishi’s story first on page 6/7 of the PDF, come together to have a discussion answering Q1, then if there is time they can read some of the other passages and move on to discuss Q2.* 

Instructions:

Consider a dialogue guided by the following questions to achieve the learning objective to relate the historical and political roots of injustice to inequity and current health care disparities. The probes and hints are optional guides for the discussion.

**Q1) Discuss the story of Ishi.**

– Were you surprised?

– How does the fact that Ishi’s death occurred at present day Parnassus campus impact you today?

– What does justice for Ishi look like?

–What do you know about the local indigenous tribes of the Bay Area?

Hint: Discuss the Ohlone Shellmounds, including the largest one at the present-day site of Bay Street Mall in Emeryville.  For reference check out:

<https://sogoreate-landtrust.org/lisjan-history-and-territory/>

**Q2) Discuss what actions can you take to acknowledge this local history as an individual? As a community? As an institution?**

–recall what Bree Newsome Bass did in 2015… <https://www.youtube.com/watch?v=dq9gYrcledU>

–Did you know 20 acres of UCSF land at Mount Sutro were demanded in 2020 to be rematriated to the Ramaytush Ohlone tribe?

Note: We picked a few examples to discuss collective and generational trauma, looking at past and present cases of what some call medical torture. The case of Elmer Allen is a local example from our own institution. The UCSF community employs a current dermatology faculty that had a part in the case of Holmsburg Prison led by Kligman.

Facilitator Guide: Small Group Activity 2 – cases (20 min)

*Note,* *each group will have a different case (see table at the end of this document) to discuss.*

Instructions:

Consider a dialogue guided by the following questions to achieve the learning objective to connect collective trauma, generational trauma, and inequities in health care, to relate with confidence examples of cases of racism in medicine to current health disparities, and to critique policies that are perpetuating health disparities. The probes and hints are optional guides for the discussion, let the participants guide the discussion.

**Q1) What may or may not protect you, your family, your neighbors, your community, from similar situations to the case you reviewed?**

Probe: If you were not protected, how do you strive to protect yourself?

- Hint: consider what behaviors help to navigate health care systems

-Hint: consider generational trauma, collective trauma, and iatrophobia, and their impact on the health trajectory of a patient

*Make sure the group moves onto the second question with at least 10 minutes remaining because the second question will be shared back in the large group with the purpose of everyone learning about all the cases*. *Try to ensure they have picked a speaker to share to the large group a 2-minute answer to Q2.*

**Q2) What is the takeaway message you would share with the large group from your case? (choose a speaker to share your answer)**

Probe – White supremacy ideology and colonization influence the health care system. Discuss how that relates to your case example.

-Hint: topics to discuss can include the health care delivery inequities due to for-profit system, employer-based insurance, underfunded “safety net” (i.e., IHS), use of standardized testing (i.e., MCAT) and AOA that try to maintain homogenous physician population.

Probe - discuss Race and Medicine.

-Hint: are some bodies worth more than others? Recall the story of Ishi, for example, from activity 1.

References for Roots and Further Reading:

i. History Review Activity PDF.
ii. Moore, E., Montojo, N., & Mauri, N. (2019). Roots, Race, & Place: A History of Racially Exclusionary Housing in the San Francisco Bay Area. UC Berkeley: Othering & Belonging Institute. Retrieved from https://escholarship.org/uc/item/2j08r197
iii. Zinn, Howard. *A People's History of the United States: 1492-Present*. Rev. and updated ed. New York: Harper Perennial, 1995. Print.
iv. Dunbar-Ortiz, Roxanne. An Indigenous Peoples’ History of the United States. Beacon Press, 2014.
v. Madley, Benjamin. An American Genocide: the United States and the California Indian Catastrophe, 1846-1873. First paperback edition., Yale University Press, 2017.
vi. Washington H. *Medical Apartheid: The Dark History of Medical Experimentation on Black Americans from Colonial Times to the Present.* New York: Anchor Books; 2006.
vii. Marya R, Patel R. Inflamed: Deep Medicine and the Anatomy of Injustice. New York: Farrar, Straus and Giroux; 2021.
viii. Luiselli, Valeria. *Tell Me How It Ends: An Essay in Forty Questions*. Minneapolis: Coffee House Press, 2017. Print.
ix. Glaude, Eddie S. *Democracy in Black: How Race Still Enslaves the American Soul*. First edition. ed. New York: Crown Publishers, 2016. Print.
x. Wilkerson, Isabel. Caste: The Origins of Our Discontents. Random House, 2020.

Note: With the recent Open Notes policy that permits patients to view medical documentation, we are giving even more reason to work together on these objectives.

Note: Oppression, for Freire, is “any situation in which ‘A’ objectively exploits ‘B’ or hinders his and her pursuit of self-affirmation as a responsible person.” Violence occurs when those who exploit fail to see others as persons. In this self-other relationship, Freire most frequently says the oppressed are treated like inanimate things rather than human beings.

Facilitator Guide for Small Group Activity:

- *Remember the purpose of the dialogue is to achieve the objectives of the session*
- *Remind participants that they can turn off video at any time for any reason and welcome everyone into the dialogue using our Agreement*
- *During introductions, invite participants to share name, site, and how they move through the world (alluding to any social identities they want to name). “my name is Yalda Shahram, I am faculty at DHM, and I move through the world as (identify as) a cis-gender woman, an immigrant, bilingual, heterosexual, person of color.”*
- *During introductions also invite participants to review the Agreement to dialogue presented in the intro slides and to add/modify it if they would like (but do not take more than 2 minutes on this).*

Instructions: There will be approximately 30 minutes for this activity. Move on to Q4 and Q5 after 10 minutes maximum.

1. **Review the language in the note with your group**
   *This is just one current example that highlights the stigma of using “poor” control, copied and pasted, even though the patient now has excellent control with a lower A1c. The use of race to describe the patient is inappropriate as it promotes race-based medicine. Race may be appropriately discussed in social history in describing stress, experiences of discrimination, etc.*
2. **Discuss how you felt about the person whom the note was about.** *Facilitators can cite the study on stigmatizing language and sickle cell disease resulting in negative attitudes and different opioid prescribing patterns by providers towards patients.*
3. **What changes would you make to humanize the language in the note?** *Emphasize the importance to change the practice of documenting or speaking about patients as inanimate things rather than human beings. Using first person. Avoiding stigmatizing words.*
   *An advanced topic to consider for this or the previous question is “othering and belonging” with reference to Edward Said’s work discussing the “making of the Other” in colonialism and imperialism. The “Other” will be discussed more in the next session on humility.*
4. **Share personal examples of documentation or oral presentations when dehumanizing language was used.** *Consider preparing one example to show that we are working together to unlearn the health care systems stigmatizing and dehumanizing language, including facilitators. In a health care system that falls short to provide equitable outcomes, experience, access and resources, the use of dehumanizing language removes emotion for the people and accountability to change the systemic oppression, defined as policies, economic systems, and institutions that produce and maintain social inequities.*
5. **How have you or might you respond when a learner or colleague uses dehumanizing language?** *Guide participants to choose to invite the learner or colleague into a dialogue about the use of dehumanizing language. Point out that language is constantly changing and we are all lifelong learners together.*

References for Unlearn:

i. Collaborative Table Link: <https://docs.google.com/document/d/1RkWM0KVvs32wv5GYaQqlFjxQH7dzb8I5/edit?usp=sharing&ouid=106093260451044034427&rtpof=true&sd=true>
ii. Words Matter: Reframing Biased Language within Information Technology. June, 2021. Shohreh Bozorgmehri, UC Irvine | shohreh@uci.edu, Divisional Director, Student and Academic Services. PDF.
iii. Goddu, Anna P., O’Conor, Katie J., Lanzkron, Sophie. et al. “Do Words Matter? Stigmatizing Language and the Transmission of Bias in the Medical Record”. *Journal of General Internal Medicine*. 2018;33: 685.
iv. Kelly, John F., and Cassandra M. Westerhoff. “Does It Matter How We Refer to Individuals with Substance-Related Conditions? A Randomized Study of Two Commonly Used Terms.” The International Journal of Drug Policy, vol. 21, no. 3, Elsevier B.V, 2009, pp. 202–07, https://doi.org/10.1016/j.drugpo.2009.10.010.
v. Kelly, John F., et al. “Does Our Choice of Substance-Related Terms Influence Perceptions of Treatment Need? An Empirical Investigation with Two Commonly Used Terms.” Journal of Drug Issues, vol. 40, no. 4, SAGE Publications, 2010, pp. 805–18, https://doi.org/10.1177/002204261004000403.
vi. Deber, Raisa B., et al. “Patient, Consumer, Client, or Customer: What Do People Want to Be Called?” Health Expectations: an International Journal of Public Participation in Health Care and Health Policy, Accepted for publication 19 August 2005, vol. 8, no. 4, Blackwell Science Ltd, 2005, pp. 345–51, https://doi.org/10.1111/j.1369-7625.2005.00352.x.
vii. Finucane, T. E., and J. A. Carrese. “Racial Bias in Presentation of Cases.” Journal of General Internal Medicine: JGIM, vol. 5, no. 2, 1990, pp. 120–21, https://doi.org/10.1007/BF02600511.
viii. Glassberg, Jeffrey, et al. “Among Emergency Physicians, Use of the Term ‘Sickler’ Is Associated with Negative Attitudes Toward People with Sickle Cell Disease.” American Journal of Hematology, vol. 88, no. 6, Wiley Subscription Services, Inc, 2013, pp. 532–33, https://doi.org/10.1002/ajh.23441.

Note: the following is an advanced organizer – a visual representation of the three components of cultural humility. This visual should be exceedingly familiar to you, and although it was created before the COVID pandemic, it is all the more relevant now.

Note: First, Let Go of the false sense of security that stereotyping brings. Pump the soap, the stereotypes.

Note: Next, listen to the water, listen to the person. The person is the only individual uniquely qualified to help you understand the intersection of race, ethnicity, religion, class, and so on informing their identity. Listen to the patient to clarify the relevance and impact of the intersection of their social identity on their present illness or wellness experience.

Note: Lend your hands to the water, lend your power to the patient, lend your authority and relinquish the role of expert to the patient, become the student of the patient **with conviction** and **explicit expression** of the patient’s potential to be a **capable**, **full** and **empowered** partner in the therapeutic alliance (hold hands together)

Note: Name the three components of practicing cultural humility. Just like hand washing, we should be reminding ourselves to do this before every single encounter. Let Go of the false sense of security stereotyping brings. Listen to the other. Lend your power, give autonomy, and become the student of the other.

Facilitator Guide:

- *Remember the purpose of the dialogue is to achieve the objectives of the session*
- *Remind participants that they can turn off video at any time for any reason and welcome everyone into the dialogue using our Agreement*
- *During introductions, invite participants to share name, site, and how they move through the world (alluding to any social identities they want to name). “my name is Yalda Shahram, I am faculty at DHM, and I move through the world as (identify as) a cis-gender woman, an immigrant, bilingual, heterosexual, person of color.”*
- *During introductions also invite participants to review the Agreement to dialogue presented in the intro slides and to add/modify it if they would like (but do not take more than 2 minutes on this).*

*Instructions: You will have a 20-minute small group dialogue guided by questions 1-3, then we will reconvene as a large group for a brief 10 minute share and break out again into our same small groups for 20 minutes to share answers to question 4.*

1. Imagine yourself as the patient. What part of your cultural identity can be misinterpreted by your health care provider?
   a) How would that impact your patient experience? Your trust for your providers?
2. How would you want to be asked about your identity by your health care provider? By your peers?
   a) How much of yourself do you share intentionally, and non-intentionally?
3. How do you ask patients about their identify? How do you ask peers? [practice asking each other with role play]
4. Describe how you can apply cultural humility to an example of cultural mismatch with a patient or peer. [you may use the example you provided in the pre-session survey or a different example]

Helpful hints and suggestions while facilitating:

*Manage time to spend about 5-7 minutes per question (1-3). Below are some examples of answers to share or you can share your own answers. These examples do not need to be shared in the small group if the participants are sharing their own, but if they are struggling or the dialogue ends early please refer to these examples:

*One possible example for Q1: As a patient, my health care provider can misinterpret my religion, my national origin, my language preference. This can fracture my trust if my provider makes assumptions instead of asking.

*One possible example for Q2: I would want to be asked directly and clearly including the motivation for why the person is asking. I am intentional (even when I do not want to be) about sharing my immigration status and national origin because my outward appearance and name have historically been automatic triggers of questions about my identity. [once in front of my team when I was on teaching wards a consultant asked me about my national origin and went on to guess a country, incorrectly]

* One possible answer for Q3:

“What’s something important about your [cultural]/[social] identity that you want me to know?”

**After 20 minutes, we will be rejoining the large group to share take aways from each group. Identify one person to share any take aways [or if anonymity wants to be upheld the facilitator may offer to be the voice of the group]. Ask the group if you can share their answer to Q3 in the chat feed as well. The 10 min large group discussion is an opportunity for learning from each before breaking back out to the small groups to discuss a specific example of cultural mismatch.

*Small groups will have 20 min to discuss and share their examples of cultural mismatch, either personal or witnessed experiences, and how to apply the practice of cultural humility to the scenario, and how to teach cultural humility using the scenario.

*If possible, encourage participants to consider at least one shared experience that is about race discordance given the significant racial disparities we have measured, in particular with pain management

References for Humility:

i. Tervalon, M., and J. Murray-García. "Cultural Humility Versus Cultural Competence: A Critical Distinction in Defining Physician Training Outcomes in Multicultural Education." *J Health Care Poor Underserved* 9.2 (1998): 117-25. Print.
ii. Rambachan, Aksharananda, et al. “Racial Disparities in 7-Day Readmissions from an Adult Hospital Medicine Service.” Journal of Racial and Ethnic Health Disparities, 2021, https://doi.org/10.1007/s40615-021-01088-3.
iii. Holmes, S. M., Hansen, H., Jenks, A., Stonington, S. D., Morse, M., Greene, J. A., Wailoo, K. A., Marmot, M. G. & Farmer, P. E. (2020). Misdiagnosis, mistreatment, and harm: When medical care ignores social forces. The New England Journal of Medicine, 382(12), 1083–1086 doi:10.1056/NEJMp1916269

Note: we invite everyone to go to the Project Implicit website to take one of these four IATs. Share the example of **internalized oppression**: “There is nothing more painful to me at this stage in my life than to walk down the street and hear footsteps . . . then turn around and see somebody white and feel relieved.”-the Rev. Jesse Jackson in the 1980.

Most white respondents tended to associate blackness with criminality.

Responses from persons who self identified as Black [in Project Implicit] were more evenly mixed, "approximately even numbers of black respondents showing a pro-white bias as show a pro-black bias.”

Note: After returning from taking the IAT, we present the work of Patricia Devine. To mitigate the habit of prejudice, we will be using evidence-based methodology.

Note: Devine came up with the idea of thinking of prejudice as a habit.  the premise that implicit bias is like a

habit that can be reduced through a combination of **awareness** of implicit bias, **concern** about the

effects of that bias, and the application of **strategies** to reduce bias.

Goff is an expert in bias and researches criminal justice reform. He and other experts created (next slide)

Note: Science of Equality, available on the online community in Teams. We will only be beginning the process for breaking habits today so I recommend everyone to take the time to check out the resources online.

Note: These are the five strategies that evidence has shown to reduce IAT scores. Remember that breaking a habit requires a combination of **awareness** and **concern** about the effects of that bias, and the application of **strategies** to reduce bias. I will review two strategies.

Note: We discuss a slide on privilege to share the link between implicit bias and dominant identities. Often, privilege can be measured by the paucity of a negative experience – for example, a person of color may have many experiences of being followed or harassed when shopping, OR the experience of feeling unsafe when being stopped by police while driving because of your race, ethnicity, or gender. OR the experience of ever been asked if you were born in the us or asked to “go back to your country”.

And it has been historically those who are dominant that have shaped the policies which today we know **as structural oppression**. Not only do individuals need to begin reforming their behaviors, but structural reform and change must also occur (next slide)

Note: Implicit bias within health care is informed by implicit bias outside of health car:

- Criminal justice, Climate justice, Housing justice – Policy
- Judiciary - Law
- Education – resources, content of textbooks (dominant narrative)
- Media – many examples, here is one measure: While African-Americans make up 29 percent of the nation's poor, they constitute 62 percent of the images of the poor in the leading news magazines, and 65 percent of the images of the poor on the leading network television news programs.

Biases of the individuals informs the media, governmental policy, law and education, leading to institutional inequity and structural oppression. We have to begin breaking the predjudice habit at the individual and institutional levels.

Facilitator Guide:

- *Remember the purpose of the dialogue is to achieve the objectives of the session*
- *Remind participants that they can turn off video at any time for any reason and welcome everyone into the dialogue using our Agreement*
- *During introductions, invite participants to share name, site, and how they move through the world (alluding to any social identities they want to name). “my name is Yalda Shahram, I am faculty at DHM, and I move through the world as (identify as) a cis-gender woman, an immigrant, bilingual, heterosexual, person of color.”*
- *During introductions also invite participants to review the Agreement to dialogue presented in the intro slides and to add/modify it if they would like (but do not take more than 2 minutes on this).*

Instructions: You will have a 20 minute small group dialogue guided by the scenario below. Reminder that break out rooms are never recorded.

Scenario: you are on a committee tasked with reviewing submissions for naming a new research building on campus. you have no information about the donors for the building.

All participants on the committee are of similar rank. All self-identify as White, except for one participant.   
This is the submission they are reviewing:

We are a collective of individuals from the Bay Area community and propose naming this new research building after Elmer Allen, in addition to providing funding for the Elmer Allen Legacy non-profit organization. The living family of Elmer Allen have requested our support for this proposal. This is the story of Elmer Allen: he was a man from Richmond who identified as Black and was part of the racist history of medical experimentation. At UCSF in the 1940’s, he was injected with Plutonium and lost his leg. The historical and political context is this: “The city of Richmond [is the] site of several major shipyards during WWII. Many blacks migrated to the area for wartime jobs but found themselves unemployed and underemployed during the postwar demobilization and deindustrialization. Much of the postwar black community lived in ghettos consisting of public housing units built by the federal government during the war. North Richmond, a town of six thousand people stuck between a garbage dump and the toxic-fume-producing Chevron Oil refinery, was almost entirely black. As an unincorporated area, the community received no public services from the city.” (Bloom J, Martin WE, 1951. *Black Against Empire: The History and Politics of the Black Panther Party.* Oakland: University of California Press; 2016.) 

Role play for the next 15 minutes the dialogue you think would take place in accepting or rejecting this submission for naming the new research building and direct funding. Below are just suggestions for the script, feel free to come up with your own lines.

- “I had not heard of this Elmer Allen person, and would want to review the evidence in the experiment they are alleging as racist, is this even real? And this community group is definitely not one of the major donors of the building, so why are we wasting our time with this submission? Reject and next.”
- “I agree, especially because naming this prestigious new building after someone like Elmer Allen may lead to losing donors and alienating for the majority of researchers who are supposed to work in there. What if they decide to move their research elsewhere? Let’s find a more safe name.”

*>>Take turns practicing how to respond. Use the Calling In/Calling Out tools (PDFs). Discuss individual and institutional bias, and the strategies to begin to break the prejudice habit.*

Session References:

i. Haslam, R.E. (2019). Interrupting Bias: Calling Out vs. Calling In. Seed the Way LLC. http://www.seedtheway.com/uploads/8/8/0/0/8800499/calling_in_calling_out__3_.pdf.
ii. Devine, Patricia G et al. “Long-term reduction in implicit race bias: A prejudice habit-breaking intervention.” Journal of experimental social psychology vol. 48,6 (2012): 1267-1278. doi:10.1016/j.jesp.2012.06.003
iii. powell, J. A, Tropp, L. R, Goff, P. A, & Godsil, R. D. (2013). The Science of Equality Volume 1: Addressing Implicit Bias, Racial Anxiety, and Stereotype Threat in Education and Health Care. UC Berkeley: Othering & Belonging Institute. Retrieved from https://escholarship.org/uc/item/2t95b844

Note: We have all been taught to practice with empathy – some describe empathy as “putting yourself in someone else’s shoes”. **Radical empathy is putting in the work to educate oneself and to listen with a humble heart to understand another’s experience from THEIR perspective, NOT as we imagine WE would feel. Radical empathy is not about you and what you think YOU would do in a situation you have never been in and perhaps never will.**

Note: In the forum theatre method of Theatre of the Oppressed, participants create a play where the protagonist (oppressed) and the antagonist (oppressor) have an encounter, with bystanders present (ally to the oppressed and ally to the oppressor), and the outcome showing that the system of oppression is maintained. This is visually depicted by the following image created by Dimir Viana and translated by Fernanda Dias. Begin by pointing to the protagonist and antagonist characters, explain that the protagonist is the oppressed and the antagonist the oppressor. The oppressed and oppressor each have their respective allies. The outcome of the interaction between the oppressed and oppressor results in the oppressor winning with no solution met. In Forum Theatre, the audience are “spect-actors” and have an active role to propose alternatives to the outcome. The scene can be repeated multiple times with audience participation.

Theatre of the Oppressed Virtual Activity Instructions:

- 4 participants break out to write their scenario for forum theater for 30 minutes. They can create any scenario that includes 4 characters: one oppressor, one oppressed, one ally of the oppressor, one ally of the oppressed. There needs to be an encounter between the oppressor and oppressed that results in the oppressor winning. Meanwhile the other participants will have large group dialogue on structures of oppression (can use shared word doc to prepare for the forum theatre)
- After 30 minutes, the group reconvenes and the actors perform the play without participation from audience. The actors will change their names on Zoom to “Protagonist/Oppressed”, “Antagonist/Oppressor”, “Ally to Oppressed”, “Ally to Oppressor”. The script will be placed into the chat feed. There will be a short monologue by the protagonist explaining their situation followed by an encounter with the Antagonist. The dialogue between them will show the protagonist actively trying to change their situation and the antagonist reacting to that action with the sequence ending with tension rising and the antagonist “winning.”
- The audience are the “spect-actors” and will be asked to take notes using the shared google doc about the problem and solution/alternative outcome during performance of the scenario. After performing the scenario once, the scenario will be repeated this time with the spect-actors (audience) called to bring an alternative outcome to the play by inserting themselves at any point into the encounter. The antagonist will read the same lines (in the chat feed) but the spect-actor who is taking the place of the protagonist may propose new lines which lead to an alternative outcome. Can insert new ally roles as well and repeat the scene. Will repeat this activity as time permits with multiple people inserting themselves into the play, proposing alternative solutions.
- Debrief – save at least 15 minutes after forum theater for debrief. This activity is built for reflection followed by action to reshape the dialogue and generate change. What action plan is called for to dismantle structures of oppression? What are the next steps? Who needs to be at the table to make policy change? This is the tip of the iceberg and we invite participants to continue the community of practice and stay connected on the online platform.

References for session:

i. Boal, Augusto. Theatre of the Oppressed. New York: Theatre Communications Group, 1985. Print.
ii. Midha, Gopal. "Theatre of the Oppressed a manual for educators." (2010).
iii. Jones, CP. (2020). Seeing the Water: Seven Values Targets for Anti-Racism Action. Harvard Medical School Primary Care Blog. Retrieved from http://info.primarycare.hms.harvard.edu/blog/seven-values-targets-anti-racism-action

(3) Literature used in multiple sessions:

1. Bracho, America, et al. *Recruiting the Heart, Training the Brain: The Work of Latino Health Access*. Berkeley, California: Hesperian Health Guides, 2016. Print.
2. Chetty, R., et al. "The Association between Income and Life Expectancy in the United States, 2001-2014." *JAMA* 315.16 (2016): 1750-66. Print.
3. Davis, Angela Y., and Frank Barat. *Freedom Is a Constant Struggle: Ferguson, Palestine, and the Foundations of a Movement*. Chicago, Illinois: Haymarket Books, 2016. Print.
4. Felitti, V. J., et al. "Relationship of Childhood Abuse and Household Dysfunction to Many of the Leading Causes of Death in Adults. The Adverse Childhood Experiences (ACE) Study." *Am J Prev Med* 14.4 (1998): 245-58. Print.
5. Glaude, Eddie S. *Democracy in Black: How Race Still Enslaves the American Soul*. First edition. ed. New York: Crown Publishers, 2016. Print.
6. Jee-Lyn García, J., and M. Z. Sharif. "Black Lives Matter: A Commentary on Racism and Public Health American Journal of Public Health. 105, e27_e30, <https://doi.org/10.2105/AJPH.2015.302706>
7. Luiselli, Valeria. *Tell Me How It Ends: An Essay in Forty Questions*. Minneapolis: Coffee House Press, 2017. Print.
8. Messer, L. C., et al. "The Development of a Standardized Neighborhood Deprivation Index." *J Urban Health* 83.6 (2006): 1041-62. Print.
9. Tervalon, M., and J. Murray-García. "Cultural Humility Versus Cultural Competence: A Critical Distinction in Defining Physician Training Outcomes in Multicultural Education." *J Health Care Poor Underserved* 9.2 (1998): 117-25. Print.
10. Zinn, Howard. *A People's History of the United States: 1492-Present*. Rev. and updated ed. New York: Harper Perennial, 1995. Print.
11. Freire, P. (1972). Pedagogy of the oppressed. New York: Herder and Herde.
12. Freire, Paulo, and Paulo Freire. Education for Critical Consciousness. New York: Seabury Press, 1973. Print.
13. Wilkerson, Isabel. Caste: The Origins of Our Discontents. Random House, 2020.
14. Goddu, Anna P., O’Conor, Katie J., Lanzkron, Sophie. et al. “Do Words Matter? Stigmatizing Language and the Transmission of Bias in the Medical Record”. *Journal of General Internal Medicine*. 2018;33: 685.
15. Cruess RL, Cruess SR, Steinert Y. Medicine as a Community of Practice: Implications for Medical Education. *Academic medicine*. 2018; 93:185-191
16. UCSF Antiracism Initiative by the Chancellor: [link](https://chancellor.ucsf.edu/blog/ucsf-anti-racism-initiative)
17. Diaz T, Navarro JR, Chen EH. An Institutional Approach to Fostering Inclusion and Addressing Racial Bias: Implications for Diversity in Academic Medicine. Teach Learn Med. 2019 Sep 28; 1-7
18. Vela MB, Kim KE, Tang H, Chin MH. Innovative Health Care Disparities Curriculum for Incoming Medical Students. Journal of General Internal Medicine. 2008; 23:1028-1032.
19. Holmes, S. M., Hansen, H., Jenks, A., Stonington, S. D., Morse, M., Greene, J. A., Wailoo, K. A., Marmot, M. G. & Farmer, P. E. (2020). Misdiagnosis, mistreatment, and harm: When medical care ignores social forces. The New England Journal of Medicine, 382(12), 1083–1086 doi:10.1056/NEJMp1916269
20. Smedley BD, Stith AY, Nelson AR eds. Unequal Treatment: Confronting Racial and Ethnic Disparities in Health Care. Committee on Understanding and Eliminating Racial and Ethnic Disparities in Health Care. *Institute of Medicine*. Washington D.C.: The National Academies Press; 2003.
21. Hoffman KM, Trawalter S, Axt JR, Oliver MN. Racial bias in pain assessment and treatment recommendations, and false beliefs about biological differences between blacks and whites. Proceedings of the National Academy of Sciences. 2016;113(16), 4296–4301.
22. Washington H. *Medical Apartheid: The Dark History of Medical Experimentation on Black Americans from Colonial Times to the Present.* New York: Anchor Books; 2006.

Appendix 3:

The introductory slides with notes, including the “agreement” slide on partaking in dialogue during the Tea House Series presented at the start of every session at University of California, San Francisco.

Note: define respect in dialogue; “to work with each other”

Note: define humility in dialogue referencing the intersectionality of social identities

Note: this illustration of intersectionality is just one example to show the weight of different social identities for one individual. These weights can change over time and in different social situations.

Note: Welcome to the Tea House. Welcome back if you have joined us in prior sessions.

Note: Our series is called the Tea House because of this cultural connotation.

Note: We review our Series objectives…

Note: We expect you to engage in the Tea House and ask for your curiosity and commitment.

Note: If at any time you need to take a break due to the nature of the content of our series, please excuse yourself and turn off your video, we understand and support you on this challenging journey.

Note: every session has its own agenda, or “roadmap” slide, with unique small group activity or activities. The first two bullet points take between 10-20 minutes to present. A recap of the prior session(s) with a slide or two is included in bullet one prior to presenting the session objectives. For the last bullet, accountability, the Microsoft Teams platform for the online community is shared on screen with a brief review of how to share a post and tag the community members.

Note: you are invited to open the Microsoft Teams Tea House and share a new post with your SMART goal. SMART = Specific, Measurable, Achievable, Relevant, and Time-Bound. On the online platform, you can also access further reading, share other resources, and support the community to take action on an individual, institutional and structural level.

Note: In each session I highlight one song/artist, referencing the social power of music. The above slide is for the Unlearn session, presented in December, because Human Rights Day is 12/10. The artist Suni Paz is part of the Nueva Cancion including other artists like Victor Khara, and many other Latin American artists. Consider the prisoners whose words caused their imprisonment, and the fatality of language for some. Other song/artists we highlight: “Old Jim Crow”/Nina Simone, Roots session; “No Bem Falá”/Lura, Humility session; “Afro Blue”/Abbey Lincoln, Habits session; “The Revolution Will Not Be Televised”/Gil Scott-Heron, Antiracist session.
